# Influence of Serotonin Transporter Gene Polymorphisms and Adverse Life Events on Depressive Symptoms in the Elderly: A Population-Based Study

**DOI:** 10.1371/journal.pone.0143395

**Published:** 2015-11-23

**Authors:** Annalisa Davin, Maria Cristina Monti, Letizia Polito, Roberta Vaccaro, Simona Abbondanza, Marco Gnesi, Simona Villani, Antonio Guaita

**Affiliations:** 1 ”Golgi Cenci” Foundation, Abbiategrasso, Italy; 2 Section of Biostatistics and Clinical Epidemiology, Department of Public Health, Experimental and Forensic Medicine, University of Pavia, Pavia, Italy; 3 “C. Golgi” Geriatric Institute, Abbiategrasso, Italy; UTHSCSH, UNITED STATES

## Abstract

**Background:**

Depression is common in the elderly. The role of genetic and environmental factors in modulating depressive symptoms is not clear.

**Methods:**

We evaluated the influence of serotonin transporter gene polymorphisms and recent adverse life events on depressive symptoms in an elderly Italian population. We used data from “InveCe.Ab”, a population-based study of 1321 subjects aged 70–74 years. We used the 15-item Geriatric Depression Scale (GDS) to assess depressive symptoms–a GDS score ≥5 points (GDS≥5) indicated the presence of clinically relevant symptoms–and performed 5-HTTLPR and rs25531 genotyping to obtain the triallelic polymorphism of the serotonin transporter. We used the Geriatric Adverse Life Events Scale to measure adverse life events, and logistic regression models to evaluate the role of genotype and recent adverse life events in depressive symptoms, controlling for potential confounders and independent predictors.

**Results:**

Two hundred subjects (15.76%) had a GDS≥5. The 5-HTTLPR triallelic polymorphism was significantly associated with GDS≥5. Only S′S′ carriers showed an increased risk of depressive symptoms (OR_adj_ = 1.81, *p* = .022); one extra adverse life event increased this risk by 14% (*p* = .061) independently of genotype. Other factors significantly related to GDS≥5 were: female gender (OR_adj_ = 2.49, *p* < .001), age (OR_adj_ = 1.19, *p* = .007), a history of depression (OR_adj_ = 4.73, *p* < .001), and comorbidity (OR_adj_ = 1.23, *p* = .001). One extra adverse life event increased the risk of depressive symptoms by 57% (*p* = .005) only in the L′L′ carriers, while antidepressant intake was directly related to GDS≥5 in the L′S′ carriers (OR_adj_ = 2.46, *p* = .036) and borderline significant in the S′S′ carriers (OR_adj_ = 2.41, *p* = .081).

**Discussion:**

The S′S′ genotype and recent exposure to adverse life events were independently associated with depressive symptoms. The S′S′ genotype, compared with the environment, exerted a predominant effect on depressive symptoms, suggesting that it reduces the efficacy of antidepressant therapy. We conclude that genetics may be an important risk factor for depressive symptoms in late adulthood.

## Introduction

Depression is a major health problem among the growing elderly population, estimated to affect around 17% of elderly people aged 75 years or older [1]. Late-life depression has detrimental effects not only on affected individuals and their families, but also on society, due to its impact on healthcare systems [2,3]. Indeed, late-life depression generates direct costs related to outpatient care and hospitalizations and indirect costs related to increased morbidity and mortality [3–5].

The causal mechanism underlying depressive symptoms in later life is still unclear, but socio-demographic, genetic and environmental risk factors are certainly involved [6]. Depressive symptoms in older people are more prevalent in women than in men [7] and in those with a history of depression [8]. Marital status also influences depression among the elderly: being unmarried or single is a risk factor for late-onset depression [9].

Recent genome-wide association studies have failed to identify any robust and replicable locus associated with depression [10], which suggests that specific genes may represent a risk factor for depression only under particular environmental conditions [11,12].

The human serotonin transporter gene (5-HTT), supposed to be associated with an increased risk of developing depression, has been extensively studied. Variations in 5-HTT negatively influence the response to environmental stress exposure [13], and *in vitro* experiments have shown that a 43 base pairs insertion/deletion in the promoter region of this gene (a polymorphism called 5-HTTLPR) regulates its expression [14,15]. The short allele (S), compared with the long allele (L), is associated with lower transcriptional efficiency, and related to anxiety [15], depression [16], bipolar disorder [17], post-traumatic stress disorder [18], and alcohol dependence [19]. Other studies, however, have not found an association of the 5-HTTLPR polymorphism with psychiatric disorders [20–22]. Moreover, another variant (SNP rs25531) with an A>G polymorphism located upstream of the 5-HTTLPR insertion designates a third functional allele, L_G_, whose expression is equivalent to that of the S allele and lower than that of the L_A_ allele [23,24]. Several other studies did not support these findings [25–27]. These conflicting results might depend on differences in the samples considered, the methods used to assess depression, the stressors considered, and the genotype definitions used.

Exposure to threatening life events is an environmental factor commonly associated with depressive symptoms across the adult life span [28]. However, the profile (i.e. type and frequency) of adverse life events varies with age; this applies particularly to stressful events such as financial difficulties, bereavements, interpersonal conflicts, and physical illnesses or disabilities (affecting the subject and/or family members).

Aging is often associated with bereavement and the death of a spouse is a major negative life event for an elderly adult [29]. The death of a sibling, too, can increase an older person’s fear of death and, in turn, aggravate his/her depressive symptoms [30].

Depression is commonly associated with medical illness [31–34], vision or hearing loss [35], and functional limitation or pain due to a chronic disorder [36]. The caregiving experience, too, can increase the risk of depression, particularly in those caring for a person with dementia [37,38].

Depression in the elderly, therefore, is related not only to physical decline but also to the loss of contact with close ties [39].

Since only a small proportion of individuals experiencing severe negative life events develop adverse health outcomes, such as depression, it is possible that an individual’s likelihood of developing depressive symptoms depends on other factors (personality [40], coping strategies [41], social support [42,43], genetic makeup).

The influence of environmental and genetic factors on depression changes during life. Exposure to threatening life events seems to increase the risk of depression both in younger people and in the over-70s, while genes have been shown to exert their negative effect more strongly earlier in life [44]. In the elderly, it could prove crucial not only to consider how adverse life events modulate the symptoms of depression, but also to investigate the role of genetic variations capable of influencing an individual’s response to the environment, e.g. the triallelic polymorphism of the serotonin transporter.

Few studies have investigated how the association of this triallelic polymorphism with the occurrence of adverse life events affects depressive symptoms in the elderly [45,46], and their results are conflicting.

We set out to investigate the influence of serotonin transporter gene polymorphisms and adverse life events on depressive symptoms in an elderly Italian population.

## Materials and Methods

### Population and setting

This work was conducted within the framework of InveCe.Ab (Invecchiamento Cerebrale in Abbiategrasso), an ongoing longitudinal population-based study (ClinicalTrials.gov, NCT01345110). The study design and methodology have already been detailed elsewhere [47]. For the purposes of the present study, we used data from the first (cross-sectional) phase of the InveCe.Ab study, which was carried out in 2009–2010. In that phase, 1773 individuals were eligible (born between 1935 and 1939 and resident in Abbiategrasso, a small town in the hinterland of Milan); of these, 1644 were available and 1321 were enrolled. We submitted all the participants to a multidimensional evaluation to assess social, clinical and neuropsychological aspects. In addition, the participants were asked to provide a blood sample for biological analyses and DNA extraction. Subjects with dementia diagnosed according to the Italian version of the Diagnostic and Statistical Manual of Mental Disorders IV (DSM IV-TR) [48] were included if they could adequately answer the evaluation questions (21 out of 39 subjects). All the participants were Caucasian.

### Depressive symptoms

We used the 15-item Geriatric Depression Scale—short form (GDS-15) [49,50], administered to all the participants by trained neuropsychologists, to assess depressive symptoms over the previous month. Each item required a “yes”/“no” answer. When the answer indicated the presence of a depressive symptom, one point was assigned; otherwise, no point was assigned. Thus, the GDS-15 had a score range of 0–15. In accordance with the literature, a score of four or less (GDS<5) was taken to indicate the absence of clinically relevant depressive symptoms, whereas a score of between 5 and 15 (GDS≥5) was taken to indicate the presence of such symptoms [51].

### Genotyping

We performed 5-HTTLPR and rs25531 genotyping, as previously described [52], to identify polymorphisms of the SLC6A4 serotonin transporter gene promoter. Genomic DNA (gDNA) was extracted from whole blood (Maxwell, Promega) and stored at 4°C.

To assess the 5-HTTLPR genotype, 50 ng of gDNA were amplified by polymerase chain reaction (PCR) with the following primers: forward 5′-GGCGTTGCCGCTCTGAATGC-3′, reverse 5′-GAGGGACTGAGCTGGACAACCA-3′ (PCR product size: L allele 529 bp; S allele 486 bp). PCR products were separated for two hours at 3 V/cm on 2% weight/volume agarose gel and stained with GelRed Nucleic Acid stain (Biotium).

Genotyping of rs25531 was performed by allele-specific PCR, using the following primers: forward A-allele specific 5′-ACCCCTCGCGGCATCCCCCCTGCACCCACA-3′; forward G-allele specific 5′-ACCCCTCGCGGCATCCCCCCTGCACCCACG-3′; common reverse 5′-TGGAGTCCGCGCGGGATTCTGGTGCCACCT-3′. The A or G specific amplification was loaded separately on 1% weight/volume agarose gel, stained with GelRed Nucleic Acid Stain (Biotium), and run for 1 hour at 5 V/cm. To check for possible genotype errors, two investigators independently interpreted the electrophoresis results; if their interpretations were not concordant, the specific genotype was re-tested.

The 5-HTTLPR and rs25531 genotypes were then combined (5-HTTLPR-rs25531), resulting in six genotypes: SS, SL_A_, SL_G_, L_A_L_A_, L_A_L_G_, and L_G_L_G_.

In view of the findings of previous studies that also combined 5-HTTLPR and rs25531 [53–55], the low-expressing genotypes SS, SL_G_ and L_G_L_G_ were designated as S′S′, the intermediate-expressing genotypes L_A_ S and L_A_L_G_ as S′L′, and the higher-expressing genotype L_A_L_A_ as L′L′.

The genotyping analysis was successfully performed in 1312 participants: nine did not consent to provide a blood sample.

### Adverse life events

We used the Geriatric Adverse Life Events Scale (GALES) to investigate the stressful life events and difficulties experienced by our study participants. Administered by trained personnel, GALES is a 26-item checklist designed to evaluate the occurrence of major adverse life events over the previous 12 months in elderly subjects [56]. For each confirmed event, we asked two further questions to assess the degree of stress related to the reported event and to investigate how the event affected the subject’s mood. For the purpose of the present study, however, we analyzed only the occurrence (number) of adverse life events.

### Other variables

Several other socio-demographic factors considered in this study were collected from municipal registers and from a social questionnaire administered by trained interviewers: gender, age at the beginning of the study (November 1^st^, 2009), marital status (defined as: coupled [currently married/cohabiting], single [never married] or uncoupled [separated/divorced/widowed]), and years of education.

We also considered depression history and current use of pharmacological drugs for depression (antidepressants [tricyclic, selective serotonin reuptake inhibitors and serotonin-norepinephrine reuptake inhibitors] or anxiolytic/hypnotic medications [benzodiazepines]) as ascertained by a geriatrician during the medical assessment. We defined subjects taking antidepressant drugs, with or without anxiolytic/hypnotic treatment, as under antidepressant treatment, and subjects using only anxiolytic/hypnotic medications as under anxiolytic treatment. We also considered comorbidity, as measured using the Cumulative Illness Rating Scale (CIRS), in which a five-level Likert scale is used to rate disease severity for each of 14 anatomical domains. The Comorbidity Index (CIRS-CI) represents the total number of categories (excluding the category Psychiatric/Behavioral) in which moderate or severe disease (CIRS score of 3, 4 or 5) is reported [57]. This clinical information was also collected from each participant by a geriatrician during the medical assessment.

### Statistical analysis

The baseline characteristics of the population were expressed as mean values and standard deviation (SD), for quantitative variables, or as percentages, for categorical variables. Differences between subjects with and without relevant depressive symptoms were evaluated by unpaired t test for quantitative variables and chi-square test for categorical variables (χ^2^ test). Similarly, we explored the association between the genotype of the combined serotonin transporter gene polymorphism (5HTTLPR-rs25531) and demographic or clinical features, applying a χ^2^ test for categorical or ANOVA for quantitative variables.

Unconditional logistic regression analysis, specifically a genotypic model that estimated the S′S′ and L′S′ genotype risk compared to L′L′ (reference category) was applied to assess the adjusted association of depressive symptoms with the 5HTTLPR-rs25531 combined genotype and adverse life events. For the adjusted association all the relevant predictors obtained from univariate analyses were included in the model. Adjusted odds ratios (OR_adj_, respectively) with 95% confidence intervals (95% CI) were derived as measures of effect and precision. Moreover, we investigated interactions between 5HTTLPR-rs25531 genotypes and the number of adverse life events and antidepressant drug use. We also stratified analyses by genotype, in order to highlight the size of the environmental risk components.

Allele frequencies were examined for each polymorphism to detect any significant deviation from the Hardy-Weinberg Equilibrium (HWE), using goodness-of-fit χ^2^ test when appropriate. The level of statistical significance was set at .05 (.10 for interactions only). Statistical analyses were performed using Stata 12 (StataCorp. 2011. Stata Statistical Software: Release 12. College Station, TX: StataCorp LP).

### Ethics statement

All the participants gave their written informed consent to the study. The study procedures were in accordance with the Declaration of Helsinki and the study protocol was approved by the Ethics Committee of the University of Pavia on October 6th, 2009 (Committee report 3/2009).

## Results

Slightly more than 50% of the participants were females and almost a third were single or uncoupled at the time of assessment (Table 1). The average duration of education was greater than six years.

The most frequent 5-HTTLPR genotype was LS, while around one in five participants had the SS genotype (Table 1). Most of the subjects had the rs25531 AA genotype, while GG was carried by less than 1%. Combining these polymorphisms, the low-expressing S′S′ genotype was present in 23% of the individuals. Genotype frequencies did not deviate from the HWE (*p*>.05). The mean number of adverse life events, as resulting from the administration of the GALES, was low and rarely higher than seven (Table 1).

**Table 1:** Overview of the baseline characteristics of the cohort. The summary statistics (mean and standard deviation) or the frequency distribution of each variable are shown in the overall population and by the presence/absence of clinically relevant depressive symptoms (GDS≥5 vs GDS<5).The statistical tests and p-values relative to analyses by GDS categories are also reported.

		Overall population	GDS < 5 (n = 1069)	GDS ≥ 5 (n = 200)	Test and p-value
**Gender (n = 1321)**	Female, n (%)	714 (54.05)	529 (49.49)	157 (78.50)	χ^2^ = 57.11, *p* < .001
	male, n (%)	607 (45.95)	540 (50.51)	43 (21.50)	
**Age in years (n = 1321)**, mean ± SD		72.04±1.45	71.96±1.45	72.35±1.43	*t* = -3.49, *p* = .0005
**Marital status (n = 1319)**	coupled, n (%)	885 (67.1)	743 (69.50)	116 (58.00)	χ^2^ = 19.91, *p* < .001
	single, n (%)	80 (6.07)	66 (6.17)	6 (3.00)	
	uncoupled, n (%)	354 (26.84)	260 (24.32)	78 (39.00)	
**Years of education (n = 1319)**, mean ± SD		6.76±3.35	6.84±3.31	6.66±3.31	*t* = 0.71, *p* = .48
**Number of adverse life events (n = 1297)**, mean ± SD		1.98±1.33	1.90±1.31	2.45±1.39	*t* = -5.39, *p* < .0001
**5-HTTLPR (n = 1312)**	LL, n (%)	423 (32.24)	347 (32.61)	59 (29.50)	χ^2^ *=* 4.67, *p* = .10
	LS, n (%)	649 (49.47)	535 (50.18)	94 (47.00)	
	SS, n (%)	240 (18.29)	182 (17.11)	47 (23.50)	
**rs25531 (n = 1312)**	AA, n (%)	1168 (89.02)	955 (89.76)	171 (85.50)	χ^2^ *=* 7.85, *p* = .02
	AG, n (%)	143 (10.90)	109 (10.24)	28 (14.00)	
	GG, n (%)	1 (0.08)	0 (0)	1 (0.50)	
**5HTTLPR-rs25531 (n = 1312)**	L′L′, n (%)	344 (26.22)	290 (27.26)	41 (20.50)	χ^2^ *=* 7.17, *p* = .03
	L′S′, n (%)	663 (50.53)	541 (50.85)	100 (50.00)	
	S′S′, n (%)	305 (23.25)	233 (21.90)	59 (29.50)	
**History of depression (n = 1260)**	positive, n (%)	292 (23.17)	167 (16.14)	114 (61.96)	χ^2^ = 184.92, *p* < .001
	negative, n (%)	968 (76.83)	868 (83.86)	70 (38.04)	
**Pharmacological treatment for depression (n = 1309)**	no treatment, n (%)	1027 (78.46)	891 (83.43)	108 (54.00)	*χ* = 103.96, *p* < .001
	anxiolytic treatment, n (%)	174 (13.29)	123 (11.52)	46 (23.00)	
	antidepressant treatment, n (%)	108 (8.25)	54 (5.06)	46 (23.00)	
**Comorbidity Index (n = 1309)**, score mean ± SD		2.27±1.53	2.12±1.42	2.82±1.72	*t* = -6.14, *p* < .001

Only 1269 subjects completed the GDS assessment and were therefore included in the analysis. Two hundred subjects (15.76%) showed clinically relevant depressive symptoms (GDS≥5) and they showed significant differences with respect to those without clinically relevant depression (GDS<5) (Table 1). In detail, people with relevant depressive symptoms were more frequently females, uncoupled, and carriers of the S′S′ genotype. They were more likely to have a positive history of depression and to be under pharmacological treatment for depression. They were also, on average, significantly older and presented a higher number of adverse life events and comorbidity than those without relevant depressive symptoms. We found no difference in mean years of education between the subjects with and those without relevant depressive symptoms.

Gender, age, marital status, number of adverse life events, history of depression, pharmacological treatment for depression, and comorbidity, therefore emerged as significant predictors of clinically relevant depressive symptoms in the multivariable analyses.

No statistically significant difference was observed (S1 Table) when comparing mean numbers of adverse life events recorded in the different 5HTTLPR-rs25531 combined genotypes (L′L′, L′S′ and S′S′ carriers). Similarly, we found no differences in pharmacological treatment for depression, history of depression, comorbidity, gender, marital status or mean age among the 5HTTLPR-rs25531 combined genotypes, excluding that a confounding effect may act on the association between depressive symptoms and 5HTTLPR-rs25531 genotype. The multivariable analysis (Table 2) revealed an increased risk of clinically relevant depressive symptoms in subjects presenting the S′S′ as opposed to the L′L′ genotype, after controlling for the effect of the significant predictors uncovered by the univariate tests (number of adverse life events, history of depression, pharmacological treatment for depression, comorbidity, marital status, gender and age). The greater risk found in the L′S′ carriers compared with the L′L′ carriers did not reach statistical significance.

**Table 2:** Mutually adjusted associations of depressive symptoms* (relevant vs not relevant) with genotype, adverse life events, pharmacological treatment for depression, history of depression, marital status, comorbidity index, gender and age distribution. **Adjusted odds ratios (OR**_**adj**_ **) with confidence intervals (95%CI) and p-values are reported^**.

		Adjusted OR (95%CI)	p-value
**Gender** (female vs male)		2.49 (1.62–3.84)	**< .001**
**Age** (1 year change)		1.19 (1.05–1.35)	**.007**
**Marital status**	single vs coupled	0.47 (0.18–1.20)	.115
	uncoupled vs coupled	1.09 (0.73–1.63)	.672
**Number of adverse life events** (1 unit change)		1.14 (0.99–1.30)	**.061**
**5HTTLPR-rs25531**	L′S′ vs L′L′	1.27 (0.81–2.01)	.297
	S′S′ vs L′L′	1.81 (1.09–3.01)	**.022**
**History of depression** (positive vs negative)		4.73 (3.15–7.09)	**< .001**
**Pharmacological treatment for depression**	anxiolytic treatment vs no treatment	1.80 (1.13–2.87)	**.014**
	antidepressant treatment vs no treatment	2.02 (1.17–3.47)	**.011**
**Comorbidity index** (1 point change)		1.23 (1.09–1.39)	**.001**

* Depressive symptomatology was defined using GDS: relevant = GDS≥5 points, not relevant = GDS<5 points.

^ Unconditional logistic regression analysis was applied to estimate adjusted OR with 95%CI and p-value.

Interestingly, a one-unit change in the number of adverse life events (i.e. one extra adverse life event measured by GALES) produced a 14% (borderline significant) increase in the adjusted estimate of risk of clinically relevant depressive symptoms, a result which suggests that life events may act as an independent predictor with respect to genetic background. The adjusted risk of relevant depressive symptoms in the females was more than twice that found in the males, and a one-year increment in age led to a significant increase in this risk (19%). Comorbidity, as evaluated using the CIRS-CI, increased the risk of having clinically relevant depressive symptoms by 23%. On the other hand, being single or uncoupled as opposed to being coupled did not significantly change the estimated risk of depressive symptoms. As expected, treatment with anxiolytic/antidepressant medications and a history of depression were significant predictors of clinically relevant depressive symptoms, independently of all the other factors.

There was no significant interaction between 5HTTLPR-rs25531 genotype and GALES scores (*p*_for interaction_ = .25) or antidepressant drug use (*p*_for interaction_ = .51), which excludes a role for these factors as effect modifiers.

Nevertheless, to explore the role of environmental risk, we ran the same multivariable analysis among subjects with the same 5HTTLPR-rs25531 genotype (Table 3).

**Table 3:** Within combined genotypes, mutually adjusted associations of depressive symptoms* (relevant vs not relevant) with adverse life events, pharmacological treatment for depression, history of depression, marital status, comorbidity index, gender and age distribution. **Adjusted odds ratios (OR**_**adj**_ **) with confidence intervals (95%CI) and p-values are reported^**.

		L′L′		L′S′		S′S′	
		OR_adj_ (95%CI)	p-value	OR_adj_ (95%CI)	p-value	OR_adj_ (95%CI)	p-value
**Gender** (female vs male)		2.24 (0.88–5.74)	.092	2.46 (1.33–4.55)	**.004**	3.31 (1.41–7.76)	**.006**
**Age** (one-year increment)		1.25 (0.94–1.66)	.118	1.34 (1.12–1.62)	**.002**	0.99 (0.77–1.26)	.917
**Marital status**	single vs coupled	-	-	0.17 (0.02–1.32)	.090	1.91 (0.50–7.27)	.340
	uncoupled vs coupled	1.35 (0.58–3.15)	.486	1.08 (0.60–1.93)	.807	0.88 (0.40–1.94)	.755
**Number of adverse life events** (1 unit change)		1.57 (1.15–2.14)	**.005**	1.10 (0.91–1.34)	.328	1.04 (0.79–1.37)	.770
**History of depression** (positive vs negative)		5.23 (2.14–12.79)	**< .001**	4.69 (2.61–8.43)	**< .001**	4.88 (2.22–10.72)	**< .001**
**Pharmachological treatment for depression**	Anxiolytic treatment vs no treatment	1.88 (0.67–5.28)	.228	1.97 (1.01–3.84)	**.047**	1.41 (0.55–3.63)	.473
	Antidepressant treatment vs no treatment	0.87 (0.26–2.83)	.811	2.46 (1.06–5.72)	**.036**	2.41 (0.90–6.47)	.081
**Comorbidity index** (one-point change)		1.28 (1.00–1.65)	**.054**	1.15 (0.96–1.39)	.126	1.35 (1.07–1.71)	**.012**

* Depressive symptomatology was defined using GDS: relevant = GDS≥5 points, not relevant = GDS<5 points.

^ Unconditional logistic regression analysis was applied to estimate adjusted OR with 95%CI and p-value.

The stratified analysis showed differences in the influence of adverse life events on depressive symptoms (Table 3). One extra adverse life event measured by GALES significantly increased, by 57%, the risk of clinically relevant depressive symptoms in the L′L′ carriers (*p* = .005) independently of the other factors included in the model, while one extra adverse life event did not significantly increase the risk of clinically relevant depressive symptoms among the L′S′ or S′S′ carriers only (*p*>.05). The presence of clinically relevant depressive symptoms was similar in subjects taking anxiolytic drugs and in those not receiving pharmacological treatment for depression in all genotype categories, although it was slightly greater (borderline significant difference) in the L′S′ carriers. The CIRS-CI modified the risk of GDS≥5 both in the L′L′ (borderline significant increase) and the S′S′ carriers, increasing it by 28% and 35% respectively, but the effect was not significant in the L′S′ carriers (Table 3). Conversely, clinically relevant depressive symptoms were directly related to antidepressant treatment in both the L′S′ and the S′S′ carriers, but in the latter this association was only borderline significant (Table 3). Finally, with regard to the effect of gender, the females, except in the L′L′ group, showed a significantly greater risk of clinically relevant depressive symptoms.

## Discussion

In this study, conducted in an elderly population, the prevalence of depressive symptoms among S′S′ carriers of the combined serotonin transporter gene polymorphism (5HTTLPR-rs25531) was higher than among L′L′ carriers. The significantly increased estimate of risk of clinically relevant depressive symptoms in the S′S′ versus the L′L′ carriers was independent of environmental and socio-demographic factors. Moreover, we showed a direct (borderline significant) relationship between the risk of depressive symptoms and the number of recent adverse life events. Finally, within the sub-cohorts with genetically homogeneous 5HTTLPR-rs25531 backgrounds, an extra adverse life event significantly increased the risk of relevant depressive symptoms only in the L′L′ carriers.

This study shows that genes could represent a main risk factor for depressive symptoms: in particular, we found that subjects who are homozygous for the S′ allele show an increased vulnerability to depression even in late adulthood. This result agrees with the findings of several studies, both *in vitro* and *in vivo*, which have demonstrated that a reduction of serotonin transporter gene expression is associated with depression. Since it is hypothesized that cognitive impairment may influence depressive symptoms [58,59], we performed a sensitivity analysis excluding the subpopulation with dementia: in this analysis, the S′S′ genotype remained a determinant of clinically relevant depressive symptoms (OR_adj_ = 1.79, *p* = .028). Consequently, it can be assumed that the association between depressive symptoms and being an S′S′ carrier of the combined serotonin transporter gene polymorphism is stable. Our findings also showed a slight association between the number of recent adverse life events and clinically relevant depressive symptoms in the elderly. Since the cross-sectional study design did not allow us to establish whether these events predated the onset of depressive symptoms, it is difficult to determine whether our findings are consistent with the accumulating evidence documenting a relationship between exposure to stressful life events and the onset of depression [28,60].

We showed that genetics, compared with environment, may play a predominant role in determining depressive symptoms in the elderly. Indeed, analyzing subjects with homogeneous 5-HTTLPR-rs25531 backgrounds we found that the S′S′ carriers were at higher risk of depression per se, and that environment did not constitute an additional risk, while a single extra adverse life event was significantly associated with an increased risk of clinically relevant depressive symptoms only in the L′L′ carriers. Similarly, other authors have found the influence of stressful life events on depression to vary considerably depending on prior individual characteristics such as genetic makeup [13].

The present study confirmed the importance of risk factors such as socio-demographic and clinical characteristics in the modulation of depressive symptoms in later life. The conflicting results reported in previous studies investigating the association of depression with serotonin transporter genotype and stressful life events [13,27] could be attributable to differences in the methodology used to assess depression or stressors, or in the age composition of the samples studied and the procedures used for their selection. Indeed, most studies in this field are conducted on selected populations, such as women [61], children [62], or people with affective disorders [63]. Few studies have investigated these associations in the elderly and sought to evaluate not only the effect of 5-HTTLPR, but also the functional polymorphism rs25531 [45,46,64]. Furthermore, to our knowledge, none of these studies took into account the role of crucial potential confounders such as a history of depression or use of anxiolytic/antidepressant drugs. The present study is concordant with previous research [8] in which older people with a history of depression showed a higher risk of having a high number of clinically relevant depressive symptoms than those who had no history of depression. Furthermore, we feel that our analysis of anxiolytic/antidepressant drug intake may represent a particular strength of the present study. Since anxiolytic/antidepressant drugs, generally used to treat depression, influence the serotonergic system, they will directly affect the primary outcome. Use of antidepressant drugs therefore likely plays an important role, modulating the depressive symptoms outcome. Indeed, we found use of these drugs to be more common in the S′S′ and L′S′ carriers, which seems to suggest that carrying the S′ allele, already an indicator of more severe depressive symptoms, may be related to a reduced ability of antidepressants to buffer depressive symptoms. This hypothesis is supported by more recent and exhaustive meta-analytic studies on the Caucasian population, which report a lower response to antidepressant treatment in association with the presence of the short allele [65,66].

Like other authors [67], we showed that females are at marked risk of developing clinically relevant depressive symptoms. In addition, an increase of just one year in age seemed to act as a major risk factor for depressive symptoms, and we hypothesize that this may also be linked to physical decline with aging. As several studies have reported, age is an important factor that is associated not only with a greater number of depressive symptoms, but also with a greater number of adverse life events and with increased comorbidity [28].

Contrary to published evidence [9], we found that being single/uncoupled was not associated with an increased risk of depressive symptoms. This discrepancy with literature data may be due to differences in the classification of marital status or to the fact that the potential role of this risk factor is very limited with respect to the role of other crucial determinants of depressive symptoms (triallelic polymorphism of the serotonin transporter, gender, history of depression and antidepressant/anxiolytic drug intake) included in the analyses.

Our study presents several limitations. First, our findings derived from cross-sectional analyses which may have produced weak evidence. Since our research is a prospective ongoing study, we plan to evaluate, in the near future, the role of genotype configuration and adverse life events on the incidence of depressive symptoms. Second, our use of the GDS may have introduced a misclassification; indeed, we may have overlooked a few subjects who are depressed but have no insight into their condition. Third, it was not possible to evaluate the potential existence of associations with traumatic events, personality traits and participants’ coping strategies, and we therefore considered only the number of adverse life events and not perceived stress in response to adversity. Several studies report that the effect of adverse life events may depend on the type of depressive disorder and decrease with age, in association with the use of different coping strategies [56,68]. Evaluation of stress and the emotional effects of adverse life events would require a specific study, taking into account not only the number of events and the genetic aspect, but also other factors such as depression diagnosis, age at onset of the disorder, and duration and recurrence of depression.

## Conclusion

Our data suggest that, even in late adulthood, a key role in depressive manifestations is played by genetics. In a population of elderly subjects presenting clinically relevant depressive symptoms we found that some had a genetic background making them more vulnerable and, independently, another portion of individuals was found to be more likely to be affected by life’s adversities.

In particular, the 5HTTLPR-rs25531 combined polymorphism, compared with environmental factors, was found to exert a predominant effect on depressive symptomatology and seemed to interfere with the antidepressant treatment response. Therefore, activities enhancing social support, generally helpful in depressed elderly people, may be particularly important in people carrying the short allele of the 5HTTLPR-rs25531 combined polymorphism who are particularly susceptible to depressive symptoms [69]. Furthermore, there exist multiple preventive and treatment approaches, including educational interventions, for subjects with chronic illness, as well as cognitive-behavioral interventions that are effective in the elderly, but too infrequently used by this population [70].

Assessment of genetic makeup and previous life events could enhance the approach to elderly subjects at risk of depression, helping to protect them against first onsets, recurrences or post-treatment relapses of depressive symptoms.

## Supporting Information

S1 Checklist This is the STROBE-STREGA checklist. (PDF)

S1 Dataset This is the dataset containing all data underlying the findings reported in the manuscript. (XLSX)

S1 Table Depressive symptoms, environmental and socio-demographic factors by 5HTTLPR-rs25531 combined genotypes. (DOCX)
