# Correction: Influence of Serotonin Transporter Gene Polymorphisms and Adverse Life Events on Depressive Symptoms in the Elderly: A Population-Based Study

**DOI:** 10.1371/journal.pone.0152858

**Published:** 2016-03-29

**Authors:** Annalisa Davin, Maria Cristina Monti, Letizia Polito, Roberta Vaccaro, Simona Abbondanza, Marco Gnesi, Simona Villani, Antonio Guaita

There is an error in Table 1, in the variable “Pharmacological treatment for depression.” The corresponding chi-square value is indicated as χ = 103.96, but it should read χ^2^ = 103.96.

**Table 1:** Overview of the baseline characteristics of the cohort. The summary statistics (mean and standard deviation) or the frequency distribution of each variable are shown in the overall population and by the presence/absence of clinically relevant depressive symptoms (GDS≥5 vs GDS<5).The statistical tests and p-values relative to analyses by GDS categories are also reported.

			Overall population	GDS < 5(n = 1069)	GDS ≥ 5(n = 200)	Test and p-value
**Gender (n = 1321)**	Female, n (%)		714 (54.05)	529 (49.49)	157 (78.50)	χ^2^ = 57.11, *p* < .001
	male, n (%)		607 (45.95)	540 (50.51)	43 (21.50)	
**Age in years (n = 1321)**, mean ± SD			72.04±1.45	71.96±1.45	72.35±1.43	*t* = -3.49, *p* = .0005
**Marital status (n = 1319)**	coupled, n (%)		885 (67.1)	743 (69.50)	116 (58.00)	χ^2^ = 19.91, *p* < .001
	single, n (%)		80 (6.07)	66 (6.17)	6 (3.00)	
	uncoupled, n (%)		354 (26.84)	260 (24.32)	78 (39.00)	
**Years of education (n = 1319)**, mean ± SD			6.76±3.35	6.84±3.31	6.66±3.31	*t* = 0.71, *p* = .48
**Number of adverse life events (n = 1297)**, mean ± SD			1.98±1.33	1.90±1.31	2.45±1.39	*t* = -5.39, *p* < .0001
**5-HTTLPR (n = 1312)**		LL, n (%)	423 (32.24)	347 (32.61)	59 (29.50)	χ^2^ *=* 4.67, *p* = .10
		LS, n (%)	649 (49.47)	535 (50.18)	94 (47.00)	
		SS, n (%)	240 (18.29)	182 (17.11)	47 (23.50)	
**rs25531 (n = 1312)**		AA, n (%)	1168 (89.02)	955 (89.76)	171 (85.50)	χ^2^ *=* 7.85, *p* = .02
		AG, n (%)	143 (10.90)	109 (10.24)	28 (14.00)	
		GG, n (%)	1 (0.08)	0 (0)	1 (0.50)	
**5HTTLPR-rs25531 (n = 1312)**		L′L′, n (%)	344 (26.22)	290 (27.26)	41 (20.50)	χ^2^ *=* 7.17, *p* = .03
		L′S′, n (%)	663 (50.53)	541 (50.85)	100 (50.00)	
		S′S′, n (%)	305 (23.25)	233 (21.90)	59 (29.50)	
**History of depression (n = 1260)**		positive, n (%)	292 (23.17)	167 (16.14)	114 (61.96)	χ^2^ = 184.92, *p* < .001
		negative, n (%)	968 (76.83)	868 (83.86)	70 (38.04)	
**Pharmacological treatment for depression (n = 1309)**		no treatment, n (%)	1027 (78.46)	891 (83.43)	108 (54.00)	*χ*^2^ = 103.96, *p* < .001
		anxiolytic treatment, n (%)	174 (13.29)	123 (11.52)	46 (23.00)	
		antidepressant treatment, n (%)	108 (8.25)	54 (5.06)	46 (23.00)	
**Comorbidity Index (n = 1309)**, score mean ± SD			2.27±1.53	2.12±1.42	2.82±1.72	*t* = -6.14, *p* < .001

There is an error in the Acknowledgments section. It should read: The authors are grateful to “Federazione Alzheimer Italia”, Milan, for overseeing the recruitment phase and the correct execution of the InveCe.Ab study and also acknowledge Valeria Marzagalli, Golgi Cenci Foundation, Abbiategrasso, for her contribution to the data collection and quality control.
