# Supplementary material for: Influence of Serotonin Transporter Gene Polymorphisms and Adverse Life Events on Depressive Symptoms in the Elderly: A Population-Based Study
Source: PLoS One. 2015 Nov 23;10(11):e0143395. doi: 10.1371/journal.pone.0143395 (PMC4658113; doi:10.1371/journal.pone.0143395)
Supplement: S1 Checklist — (PDF) [file pone.0143395.s001.pdf]

STROBE Statement with STREGA Reporting Recommendations

	Item No	Recommendation
**Title and abstract**	1	(*a*) Indicate the study's design with a commonly used term in the title or the abstract MANUSCRIPT TITLE
		(*b*) Provide in the abstract an informative and balanced summary of what was done and what was found MANUSCRIPT ABSTRACT
**Introduction**		
Background/rationale	2	Explain the scientific background and rationale for the investigation being reported MANUSCRIPT INTRODUCTION
Objectives	3	State specific objectives, including any pre-specified hypotheses MANUSCRIPT LINE 101-102
STREGA RECOMMENDATION		***State if the study is the first report of a genetic association, a replication effort, or both.*** MANUSCRIPT INTRODUCTION
**Methods**		
Study design	4	Present key elements of study design early in the paper MANUSCRIPT LINE 105-107
Setting	5	Describe the setting, locations, and relevant dates, including periods of recruitment, exposure, follow-up, and data collection SECTION "POPULATION AND SETTINGS"
Participants	6	(*a*) *Cohort study*—Give the eligibility criteria, and the sources and methods of selection of participants. Describe methods of follow-up<br>*Case-control study*—Give the eligibility criteria, and the sources and methods of case ascertainment and control selection. Give the rationale for the choice of cases and controls<br>*Cross-sectional study*—Give the eligibility criteria, and the sources and methods of selection of participants SECTION "POPULATION AND SETTINGS"
STREGA REQUIREMENT		***Give information on the criteria and methods for selection of subsets of participants from a larger study, when relevant.***
		(*b*) *Cohort study*—For matched studies, give matching criteria and number of exposed and unexposed<br>*Case-control study*—For matched studies, give matching criteria and the number of controls per case
Variables	7	Clearly define all outcomes, exposures, predictors, potential confounders, and effect modifiers. Give diagnostic criteria, if applicable MANUSCRIPT LINE 116-178
STREGA REQUIREMENT		***Clearly define genetic exposures (genetic variants) using a widely-used nomenclature system. Identify variables likely to be associated with population stratification (confounding by ethnic origin).*** MANUSCRIPT LINE 116-196
Data sources/ measurement	8*	For each variable of interest, give sources of data and details of methods of assessment (measurement). Describe comparability of assessment methods if there is more than one group MANUSCRIPT LINE 116-178
STREGA REQUIREMENT		***Describe laboratory methods, including source and storage of DNA, genotyping methods and platforms (including the allele calling algorithm used, and its version), error rates and call rates. State the laboratory/centre where genotyping was done. Describe comparability of laboratory methods if***

		there is more than one group. Specify whether genotypes were assigned using all of the data from the study simultaneously or in smaller batches. SECTION "GENOTYPING"
Bias	9	Describe any efforts to address potential sources of bias
STREGA REQUIREMENT		***For quantitative outcome variables, specify if any investigation of potential bias resulting from pharmacotherapy was undertaken. If relevant, describe the nature and magnitude of the potential bias, and explain what approach was used to deal with this.***
Study size	10	Explain how the study size was arrived at SECTION "POPULATION AND SETTINGS" AND TABLE 1
Quantitative variables	11	Explain how quantitative variables were handled in the analyses. If applicable, describe which groupings were chosen and why MANUSCRIPT LINE 116-178
STREGA REQUIREMENT		***If applicable, describe how effects of treatment were dealt with.***
Statistical methods	12	(*a*) Describe all statistical methods, including those used to control for confounding SECTION "STATISTICAL ANALYSIS"
		(*b*) Describe any methods used to examine subgroups and interactions SECTION "STATISTICAL ANALYSIS"
		(*c*) Explain how missing data were addressed
		(*d*) *Cohort study*—If applicable, explain how loss to follow-up was addressed<br>*Case-control study*—If applicable, explain how matching of cases and controls was addressed<br>*Cross-sectional study*—If applicable, describe analytical methods taking account of sampling strategy
		(*e*) Describe any sensitivity analyses MANUSCRIPT LINE 297-302
STREGA REQUIREMENT		***State software version used and options (or settings) chosen.*** MANUSCRIPT LINE 196<br><br>***State whether Hardy-Weinberg equilibrium was considered and, if so, how.*** MANUSCRIPT LINE 193-194<br><br>***Describe any methods used for inferring genotypes or haplotypes.***<br><br>***Describe any methods used to assess or address population stratification.***<br><br>***Describe any methods used to address multiple comparisons or to control risk of false positive findings.***<br><br>***Describe any methods used to address and correct for relatedness among subjects***
**Results**		
Participants	13*	(a) Report numbers of individuals at each stage of study—eg numbers potentially eligible, examined for eligibility, confirmed eligible, included in the study, completing follow-up, and analysed SECTION "POPULATION AND SETTINGS" AND TABLE 1
		(b) Give reasons for non-participation at each stage SECTION "POPULATION AND SETTINGS"
		(c) Consider use of a flow diagram
STREGA		***Report numbers of individuals in whom genotyping was attempted and numbers of individuals in whom genotyping was successful.*** SECTION "GENOTYPING"

REQUIREMENT		
Descriptive data	14*	(a) Give characteristics of study participants (eg demographic, clinical, social) and information on exposures and potential confounders MANUSCRIPT LINE 202-212 AND TABLE 1
		(b) Indicate number of participants with missing data for each variable of interest TABLE 1
		(c) *Cohort study*—Summarise follow-up time (eg, average and total amount)
STREGA REQUIREMENT		***Consider giving information by genotype.*** TABLE 2
Outcome data	15*	*Cohort study*—Report numbers of outcome events or summary measures over time
		Case-control study—Report numbers in each exposure category, or summary measures of exposure
		Cross-sectional study—Report numbers of outcome events or summary measures SECTION “RESULTS”
STREGA REQUIREMENT		***Report outcomes (phenotypes) for each genotype category*** TABLE 2
Main results	16	(*a*) Give unadjusted estimates and, if applicable, confounder-adjusted estimates and their precision (eg, 95% confidence interval). Make clear which confounders were adjusted for and why they were included SECTION “RESULTS” AND TABLES
		(*b*) Report category boundaries when continuous variables were categorized
		(*c*) If relevant, consider translating estimates of relative risk into absolute risk for a meaningful time period
STREGA REQUIREMENT		***Report results of any adjustments for multiple comparisons.*** SECTION “RESULTS” AND TABLES
Other analyses	17	Report other analyses done—eg analyses of subgroups and interactions, and sensitivity analyses MANUSCRIPT LINE 297-302
**Discussion**		
Key results	18	Summarise key results with reference to study objectives MANUSCRIPT LINE 282-292
Limitations	19	Discuss limitations of the study, taking into account sources of potential bias or imprecision. Discuss both direction and magnitude of any potential bias MANUSCRIPT LINE 353-361
Interpretation	20	Give a cautious overall interpretation of results considering objectives, limitations, multiplicity of analyses, results from similar studies, and other relevant evidence MANUSCRIPT LINE 293-346
Generalisability	21	Discuss the generalisability (external validity) of the study results SECTION “CONCLUSION”
**Other information**		
Funding	22	Give the source of funding and the role of the funders for the present study and, if applicable, for the original study on which the present article is based
