# Supplementary material for: Influence of Serotonin Transporter Gene Polymorphisms and Adverse Life Events on Depressive Symptoms in the Elderly: A Population-Based Study
Source: PLoS One. 2015 Nov 23;10(11):e0143395. doi: 10.1371/journal.pone.0143395 (PMC4658113; doi:10.1371/journal.pone.0143395)
Supplement: S1 Table — (DOCX) [file pone.0143395.s003.docx]

**S1 Table. Depressive symptoms, environmental and socio-demographic factors by 5HTTLPR-rs25531 combined genotypes. The appropriate test and p-value for each investigated association are reported.**

|  | | **5HTTLPR-rs25531 combined genotype** | | |  |
| --- | --- | --- | --- | --- | --- |
|  |  | **L**′**L**′ **n=344** | **L**′**S**′ **n=663** | **S**′**S**′ **n=305** | **Test and**  **p-value** |
| **Depressive symptoms** | not relevant (GDS<5), n (%) | 290 (87.61) | 541 (84.40) | 232 (79.79) | χ^2^=7.17, ***p*=.028** |
|  | relevant (GDS≥5), n (%) | 41 (12.39) | 100 (15.60) | 59 (20.21) |  |
| **Gender** | female, n (%) | 179 (52.03) | 366 (55.20) | 163 (53.44) | χ^2^=.96, *p*=.61 |
|  | male, n (%) | 165 (47.97) | 297 (44.80) | 142 (46.56) |  |
| **Age (years)**, mean ± SD | | 72.14±1.45 | 72.03±1.45 | 71.95±1.44 | F=1.47, *p*=.23 |
| **Marital status** | coupled, n (%) | 227 (65.99) | 454 (68.58)) | 198 (65.13) | χ^2^=6.55, *p*=.62 |
|  | single, n (%) | 21 (6.10) | 39 (5.89) | 19 (6.25) |  |
|  | uncoupled, n (%) | 96 (27.91) | 169 (25.53) | 87 (28.62) |  |
| **Number of adverse life events**, mean ± SD | | 1.87±1.34 | 2.06±1.35 | 1.95±1.29 | F=2.41, *p*=.09 |
| **History of depression** | positive, n (%) | 71 (21.98) | 145 (22.91) | 76 (25.76) | χ^2^=1.37, *p*=.51 |
|  | negative, n (%) | 252 (78.02) | 488 (77.09) | 219 (74.24) |  |
| **Pharmacological treatment for depression** | no treatment, n (%) | 265(78.17) | 526 (79.70) | 229 (76.08) | χ^2^=4.40, *p*=.35 |
|  | anxiolytic treatment, n (%) | 46 (13.57) | 88 (13.33) | 39 (12.96) |  |
|  | antidepressant treatment, n (%) | 28 (8.26) | 46 (6.97) | 33 (10.96) |  |
| **Comorbidity index**, mean ± SD | | 2.18±1.48 | 2.24±1.48 | 2.43±1.65 | F=2.25, *p*=.11 |
